# Milestones in Software Engineering and Knowledge Engineering History: A Comparative Review

**DOI:** 10.1155/2014/692510

**Published:** 2014-01-27

**Authors:** Isabel M. del Águila, José Palma, Samuel Túnez

**Affiliations:** ^1^Department of Informatics, University of Almería, Spain; ^2^Department of Information and Communication Engineering, University of Murcia, Spain

## Abstract

We present a review of the historical evolution of software engineering, intertwining it with the history of knowledge engineering because “*those who cannot remember the past are condemned to repeat it*.” This retrospective represents a further step forward to understanding the current state of both types of engineerings; history has also positive experiences; some of them we would like to remember and to repeat. Two types of engineerings had parallel and divergent evolutions but following a similar pattern. We also define a set of milestones that represent a convergence or divergence of the software development methodologies. These milestones do not appear at the same time in software engineering and knowledge engineering, so lessons learned in one discipline can help in the evolution of the other one.

## 1. Introduction

Software is present in every-day human activities; as Bjarne Stroustrup observed, *“our civilization runs on software*.*”* Computer applications serve as the basis for modern scientific research, contribute to solving engineering problems, assist in decision making in business, and are the key factor that differentiates modern products and services. People often leave their welfare, security, jobs, entertainment, and their own decisions in the hands of a software application. But software, as an industrial product, is invisible to most of the world, except when it fails or crashes. In order to maintain its transparency, software product must be developed by engineering methods that ensure the quality of the resulting product. Software engineering (SE) is a discipline that has evolved since it was originally proposed [1] and can now be defined as “…a discipline that adopts engineering approaches, such as established methodologies, processes, tools, standards, organization methods, management methods, quality assurance systems, and the like, in the development of large-scale software seeking to result in high productivity, low cost, controllable quality, and measurable development schedule” [2].

We present a survey of the evolution of SE, comparing it against an other computer science discipline, knowledge engineering (KE). Historically KE and SE have followed a similar pattern of evolution but along parallel paths. Summarizing the history of KE and SE is hard, as there are very many prestigious works in both disciplines [3, 4], and there is no unified timeline. We extend the initial proposal of Endres [5] and Liao [6] by defining disjoint eras and divide each one into periods, which can be overlapped. Each period has its own goals, methods, and tools, and also each one has its own challenges. Progress usually appears in terms of research long before they are stabilized. For example, client-server system appeared before 1993, but we have considered the inclusion of any new idea in the evolution when it has been extended to the research or to the commercial community.

The rest of the paper is organized in six sections. Sections 2 and 3 summarize the timeline of SE and the main methodologies. The same description for KE appears in Sections 4 and 5.  Section 6 outlines the major milestones that highlight the similarities in the evolution of both disciplines. Finally, in Section 7 we give some conclusions.

## 2. Timeline of Software Engineering

Table 1 shows the characteristics of each era and period of the SE timeline, together with the most popular methodologies. We start this timeline in 1956 because it is generally thought that the first operating system was produced in 1956 by General Motors.

### 2.1. SE Era I: Mastering the Machine (1956–1967)

The term software engineering had not yet been coined. Code development was strongly influenced by outside forces. The main purpose of any piece of software was to optimize exploitation of the limited hardware resources. The first compilers were defined; operating systems were noninteractive. These primitive environments continued evolving up to the definition of the first low-level Computer Aided Software Engineering tools (CASE tools) facilitating interactive editing, compiling, and debugging. The lack of software development methods led to high risk and the origin of a new stage is easily noticeable.

### 2.2. SE Era II: Mastering the Process (1968–1982)

The first software crisis in this stage led to the birth of software engineering [1]. The aim was to reduce risk during development and improve quality and productivity. Software development methodologies appeared to define and monitor software building. An important contribution of this stage was the formal modeling approach that enables implementation automation. But for industry, this formal approach was unfeasible due to a lack of tools and training. Furthermore, formal methods become unmanageable for large system development. In conclusion, in this stage, the need to focus on predesign phases and the use of more or less formal models for software specification began to appear. A number of structured methods, such as Software Requirement Engineering Methodology (SREM) [7] and the Structured Analysis and Design Technique (SADT) [8] were developed allowing the development of specification documents for business management software.

### 2.3. SE Era III: Mastering the Complexity (1983–1992)

The up to then dominion of hardware over software ended. Personal computers arrived and opened the fields of computer applications. The software development process would now comprehensively address analysis and design from the specification. Graphical user interface and visual programming brought software closer to customers. The use of structured family and data modeling methodologies was extended [9]. Several CASE tools facilitated software development. However, data modeling (database) and function modeling (structured methods) still followed separate paths. These two modeling paths converged in object-oriented (OO) methods like early on in structured methodologies, they were first introduced in coding and design, and finally in specification and analysis [10–12]. This approach enables efficient reuse of object-oriented software and thus improves building software productivity.

### 2.4. SE Era IV: Mastering the Communications (1993–2001)

The emergence of the Internet brought with it a new software concept. The decentralization of functions and data led to the rapid development and expansion of areas of computing, such as concurrent programming and distributed architectures, which up to then had been limited to a narrower context. In addition to client/server applications, and in general, any distributed system development, there was now a new engineering software discipline called Web engineering [13]. Moreover, software development was viewed as an industrial process in which quality should be monitored. This requires an effective separation between process and product. Some tasks related to managing and improving both the product and process appeared as new SE components, such as CMM (capability maturity model) and CMMI (capability maturity model integrated) [14].

### 2.5. SE Era V: Mastering the Productivity (2002–2010)

Most software systems created in this stage are called management information systems. They were designed to be an important part of business management in large companies. This has led to a need for the methodologies to be adapted by increasing the abstraction levels in software engineering tasks up to the abstraction level in which the problem is described. New tools enabling analysis level programming, such as Model Driven Architecture (MDA) [15], appeared in this stage. The other major significant period in this stage was marked by the emergence of agile methodologies. Agile projects focus on creating the best software to meet customer needs. This means that the development team focuses only on tasks and processes that provide the customer with added value in the product being created, improved, or implemented [16]. The most popular methodologies are Extreme Programming (XP) [17] and Scrum [18].

### 2.6. SE Era VI: Mastering the Market (2011–…)

Now, there are new platforms for integration and interoperability between different information systems. The concept of Service Oriented Architecture (SOA) coined in the early decade is widely extended. It is based on the combination of basic services (own or outside) that provide the functionality at business level for a specific domain problem. These services are orchestrated to perform more complex tasks, becoming composite services. These ubiquitous real-time services can be sold as a product, which is the origin of Cloud computing. On the other hand, customers demand several applications to be used in their smartphones, tablets, or laptops. The applications (i.e., apps) are small programs that can be downloaded and installed on mobile devices that allow users to perform some tasks at the place that they are in any moment. They are grouped into virtual stores and many of them are free, so usually they are closer to marketing challenges than software development challenges. They tend to be more dynamic than traditional programs and are the ultimate expression of agile methods and MDA.

## 3. Main SE Methodologies

Methodologies that are currently in use are the evolution and/or the unification of methodologies defined and applied in previous eras. Reporting the history of the SE methodologies was hard work outside of this paper. This section collects a brief summary of the distinctive features of several selected SE development methodologies. We identify three groups of methodologies: structured methodologies, object-oriented methodologies, and agile methodologies.  Table 2 summarizes the most relevant methodologies including the main features of structured methodologies are some of them obsolete, but with a clear influence on today's methods.

First techniques that could be called methodologies, such as SREM and SADT, extended the concepts of modularization and information hiding, previously applied in structured programming, from design to specification phase. After these initials works, Structured System Analysis and Structured System Design (SSADM) were proposed to support the tasks of analysis and design [9].

Object-oriented methodologies apply the OO programming paradigm (OOP), which came to represent in the nineties what structured programming was for the seventies. OOP defines the software as a set of objects that interact with each other. Their purpose is getting more consistent, robust, and reusable software systems.

Since the nineties, modeling is the core in all the activities executed during software development. When OO methodologies are applied, the building software process starts with the problem domain model. This model will gradually evolve towards the solution domain models, being the last model the OO code. This modeling approach was applied by a great number of OO methods. After this rush of new methodologies, conflicts began to appear between similar methods, each one with its own alternative approach. Due to this fact, rational proposed an integration of different projects led by the creators of the main methods, which led to the design of a Unified Modeling Language (UML). This integration provided interoperability to OO-based methodologies, which helped the stabilization of the OO market. The current version of UML is 2.4.1 [19].

Agile methods promote a disciplined project management process that encourages frequent inspection and adaptation. These methodologies are based on iterative development. Their basie foundations were published in the Agile Manifesto by a groups of software practitioners and consultants [20]. These focal values are: individuals and iterations over processes and tools, working out software over comprehensive documentation, customer collaborations over contract negotiations, and responding to changes over following a plan. Agile methods break the tasks into small increments with minimal planning, each one called timebox. Each iteration is a full development cycle, generating a release that needs to be demonstrated to stakeholders. A method can be considered agile when software development is incremental (small software releases, with rapid cycles), cooperative (customer and developers working together with close communication), straightforward (the method itself is easy to learn and to modify), and adaptive (able to make last moment changes).

Currently due to the popularity of mobile applications (apps), methodologies need to migrate to this new kind of software products. An app can be property of private programmers or any enterprise. They do not usually embody complex programming skills. However, programmers have to manage a wide array of screen sizes, hardware specifications and configurations because of intense competition in mobile platforms. However, there is not a new methodology for this kind of software; we believe that agile methods are a good alternative.

## 4. Timeline of Knowledge Engineering

Knowledge engineering (KE) is another computer science field that shares some of the SE objectives. KE is required when the software to be developed has to behave in a heuristic way. The goal of KE is similar to that of SE: “…constructing Knowledge Based System (KBS) in a systematic and controllable manner. This requires an analysis of the building and maintenance process itself and the development of appropriate methods, languages and tools suitable for developing KBSs” [21]. Due to the fact that the two disciplines propose building software using engineering principles, there should be similarities between the methods, techniques, and tools used in both fields. In fact, they have experimented a similar evolution but almost with a decade of delay. However, KE and SE have ignored each other, against some basic principles of any engineering (e.g., reuse, cooperation, or work partition) [2]. We summarize KE evolution in Table 3.

### 4.1. KE Stage I: Mastering Intelligence (1956–1978)

In this stage, knowledge engineering had not yet appeared. The term Artificial Intelligence (AI) was coined, although some authors such as Alan Turing (from 1900 to 1956) had previously made proposals close to what would later be called AI. AI seeks to develop systems that can “think” like human experts. During this period, most work was directed at the development of general problem-solving techniques, such as the STRIPS (Stanford Research Institute Problem Solver System) planning system [22] or GPS (General Problem Solver) [23]. But these techniques were insufficient to solve real concrete problems, since they required specific domain knowledge rather than general knowledge, for which techniques for transferring knowledge from the expert to computers are required. This vision gave birth to the first KBSs, such as PROSPECTOR [24] and MYCIN [25], without the support of any development methodology.

### 4.2. KE Stage II: Mastering the Process (1979–1989)

Due to the lack of clear methods, the transition of KBSs from research to commercial products was a failure in most cases. As no engineering method existed, the development process had to face many problems. Time and cost estimates were not satisfied, products did not meet the customers' expectations, and maintenance and testing tasks became costly. Basically, building a KBS was conceived as a process of transferring human expert knowledge to a computer knowledge base [21]. In this approach, knowledge acquisition became the most important task as well as the main bottleneck in KBS development. In the same way the software crisis resulted in establishing SE as a discipline; early KBSs development problems made clear the need for more methodological approaches and a better analysis of the process applied [26]; knowledge engineering was born. Moreover, the wide scope of applicability of Artificial Intelligence techniques drove this discipline to specialize and diversify in new disciplines such as data mining, computer vision, and pattern recognition.

### 4.3. KE Stage III: Mastering the Complexity (1990–2000)

A is an attempt to overcome the knowledge acquisition bottleneck, a new generation of methodologies in the early nineties redefined KE from transfer/mining to a modeling perspective. This approach is based on the knowledge level concept proposed by Nevell, in which a level above the symbolic level provides sufficient abstraction for the knowledge to be represented, regardless of its implementation [27]. Once KBS development was defined as a modeling process and generic knowledge models were identified, a methodology to assist in the specification of the different models was required. Based on these ideas, a second generation of KE methodologies came to light. The most commonly used were CommonKADS (Compressive Methodology for KBS Development) [28], MIKE (Model-based and Incremental Knowledge Engineering) [29], and Protégé-II [30]. They represented the first attempts to provide a complete methodology for the entire KBS development lifecycle. Moreover, as in SE, the need to improve productivity led to the empowerment of knowledge component reuse, in the same way that classes and objects are reused in object-oriented development.

### 4.4. KE Stage IV: Mastering Communications (2001–2010)

The new concept of distributed software was also extended to KBS, making it possible to apply this technology to a wider range of domains and more complex problems. During the last decade, the exponential growth of information on the World Wide Web (WWW) made the ability to understand and manage the semantics of the data of paramount importance for the successful discovery, sharing, distribution, and organization of this information. Thus new challenges, such us those related to the recovery, extraction, publication, and sharing of knowledge on the WWW, have to be confronted. Two new interrelated disciplines have emerged to help face these problems, ontological engineering [31] and Semantic Web [32]. They conceive the WWW as an intelligent network, where computers are able to understand data and then use them to infer new conclusions. In an era dominated by communications, the availability of large amounts of data about specific areas of application requires the use of machine learning and data mining techniques to make it possible for computers to learn. Such programs must be able to generalize behavior from unstructured information available in the form of examples and on the WWW [33].

### 4.5. KE Stage V: Mastering the Productivity (2010–…)

An important issue in this stage is related to making AI techniques commercially viable to extend them to a new generation of consumer products, such as interactive smart toys, or their application to specific domains in which, up to now, they had not entered, such as SE itself (i.e., Search Based Software Engineering (SBSE)) [34]. Moreover, the widening scope of software solutions covers larger, more commercially complex systems, in which the need for software systems to be able to coordinate information and knowledge management in a single product is evident. The development of this kind of software should be approached from a coordinated application of KE and SE methodologies, because products generated by both development approaches can be combined, giving end users a single view of the software product. This is the main aim of a new methodological approach called SKEngineering [35].

## 5. Main KE Methodologies

Originally, pioneering KBS development methodologies focused on acquiring knowledge. Software construction was understood as the transfer and transformation of experience in problem-solving knowledge from any source (in most cases a human expert) to computer software. According to Buchanan et al. [26], this transfer required the intervention of a knowledge engineer intermediary. The most relevant KE methods are summarized in Table 4.

Buchanans main contribution was identification of knowledge acquisition as the KBS development bottleneck. Buchanan proposed a knowledge acquisition lifecycle covering all the steps in system development, that is, from initial system definition to its maturity. It was the first attempt at a commercial KBS development approach. During the following years, many changes and improvements in methodologies were promoted. As a result, new methodologies developed KBS from an SE perspective, KLIC (Knowledge Based System Life Cycle) [36], and IDEAL [37].

In the early nineties, when KE went from knowledge transfer to knowledge modeling, second-generation methodologies enable analysis of the system at knowledge level. This new approach makes it possible to specify the problem at different granularity levels and define reusable knowledge components. These methodologies are based on the modeling approach, which has its roots in previously proposed ideas, but which were, however, still far from being considered methodologies [38, 39]. KADS (Knowledge Acquisition and Design Structuring) and Protégé were the first projects addressing the problem of knowledge acquisition from the modeling point of view. Based on these projects, more complete methodologies have been developed, among which, are CommonKADS [28], MIKE [29], and Protégé-II [30].

The last group of KE methods are ontology engineering. Ontological engineering refers to all the activities that concern ontology development: ontology life cycle, methods and methodologies for building ontologies, and tool suites and languages that support them [31]. We considered Cyc as the oldest ontological engineering project [40]. A more formal approach is used by TOVE (Toronto Virtual Enterprise) Project. It uses a first order logic approach to represent ontologies in business integration architectures [41]. METHONTOLOGY is the best known ontology development methodology and the most complete and detailed of the development processes, proposing a process model, an ontology lifecycle, and a specification for all activities. These activities are classified into three categories: management, development/building, and support and they allow ontologies to be built at the knowledge level, using IEEE Standard 1074-1995. An other method based on UML and use cases is UPON (United Process for Ontologies) that has an incremental and iterative lifecycle [42].

## 6. Convergence and Divergence Milestones

**Figure 1:**
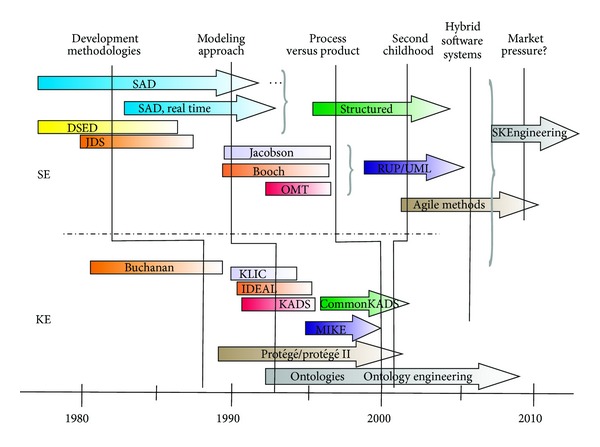
Methodologies evolution and milestones.

We can outline six major milestones in the evolution of methodologies for building software, which have a slight delay between their appearance in SE and KE. Each of those milestones is a unification moment between methodologies or is a bifurcation towards new approaches (see Figure 1). All milestones have a thin gap between their appearances in the KE and SE, and are points of inflection in the evolution of the methodologies.

The first one is the need for *development methodologies*. This milestone was the origins SE and KE, as a result of both crises. The second milestone is the migration to the *modeling approach* of; here the goal is the development of models that support the construction of software; today it is a fundamental and necessary step of all software developments. The need to evolve toward the modeling approach appears in KE faster than in SE, because KE methods are applied in more complex domains, where models become a necessity. This led to the boom of the second-generation KE methodologies.

The third milestone appears when SE and KE need to develop their projects in a more controlled way. That is, the process itself reaches the same importance that the artifacts generated during a project. The processes and the products have their own lifecycle that can be enhanced and controlled separately. The *process versus product* milestone can be clearly shown in SE evolution, but we can see in the Figure 1 that when KE methodologies reached the enough maturity to deal with this milestone, they were hit by another big change or bust that we labeled as a second crisis.

This fourth milestone (called *second childhood*) appeared in the KE due to the lack of success of the commercial software developed under KE discipline. This fact, together with the establishment and successful development of ontologies and Semantic Web, led KE back to childhood. Development efforts focus on building and publishing sets of useful concepts within a domain and the relationships between those concepts (i.e., ontology engineering). The milestone where developers returned to a second childhood has also occurred in the SE field. Agile methods appear in order to enhance the quality of the final software product by introducing checkpoints where customer's requirements can be reassigned. The agile development is a radical deviation from other software development methods, focusing on adapting the software quickly to changes in realities. It represents a big change in SE evolution.

These four milestones are points of convergence between existing methodologies, which collect and adapt the best of each method, or they are divergence points towards other applicable approaches. We propose other two milestones that are not completely stable today. *Hybrid software development* suggests that development of a software system must be treated from KE and SE points of view, by integrating the two behaviors that can be present in a software system: algorithm and heuristic [35, 43]. The common challenge must be now to integrate the best of each approach in a new holistic approach (i.e., SKEngineering). But nowadays, *market pressure* gives rise to a strong divergence milestone in SE in order to define good processes and practices (i.e., methodologies) for new approaches as Cloud computing or apps development. This milestone is a challenge for SE but for KE is yet unknown.

## 7. Conclusions

This work presents a timeline of six major eras of SE and compares that against the major development approaches of KE with the aim to search for a unified scenario to develop software systems, which represents a further step forward to understand the current state of both types of engineering. Software developers must learn about the computer science history in order to avoid divergent approaches that make our work hard when a software project is addressed.

The lack of cooperation between SE and KE can be avoided if a computer science discipline gives to the other those capabilities in which it has more experience, more potential, and more expressiveness. These interactions have been fruitful and beneficial, achieving a synergistic effect [44, 45]. For example, KE can learn from SE about the production, reuse, maintenance, and management [46], and SE can learn from KE about information acquisition techniques to improve communication with customers, or how to get specifications that best fit customers' needs [47], or how to use AI techniques in order to enhance SE process [34].

The industrial and business scenario could help to mix up modern approaches of KE and SE in order to find integration under the same shadow of techniques and methods applied in both types of engineering. This joined approach (SKEngineering) allows the development of quality products using SE or KE methods, since there are many cases in which companies require deploying software systems that integrate components based and not based on knowledge in a transparent way. Nowadays, when it is necessary to combine KE and SE methods in a project, the solution consists of doing an early separation of activities related to each discipline following each one's own path. This unified scenario should provide a reference point to support the entire software development project, which must be able to be adapted and instantiated to the development teams and the nature of the project. But there are many other challenges under study, as how KE must treat the *market pressure*. This evolution milestone can be assisted by well known Artificial Intelligence techniques as machine learning or fuzzy approaches.

## Figures and Tables

**Table 1:** Software engineering timeline.

Software engineering			
Era	Periods	Description	Methodologies
Mastering machine (1956–1967)	Batch	Hardware dependent high level languages	
	Interactive	Online. Code and fix	

Mastering process (1968–1982)	Process	Crisis. Development process software engineering	SREM, SADT DSED, JSP SSADM
	Formal	Ensure correctness. Models inapplicability in big problems	

Mastering complexity (1983–1992)	Structured	Personal computer. Expanding data and functional convergence	Modern SSADM JSD OMT Booch Jacobson
	Object oriented	Reusing new programming approach	

Mastering communications (1993–2001)	Industrial	Internet. Client/server complex projects	CORBA RUP/UML
	Distributed	Integrated methods quality	

Mastering productivity (2002–2010)	Abstraction	Conceptual level expansion Customer productivity Customer involvement	MDA XP Scrum
	Agile		

Mastering market (2011–…)	Service	Outsourcing services Orchestrating services Market demands. Downloads	BPMN/BPEL SOA-Cloud APP
	Mobility		

**Table 2:** SE methodologies summary.

Artefact	Notation	Stages/workflows
Structured system analysis and design methodology (SSADM) [9]		
Requirement specification Analysis model Design model	Data flow diagrams Data dictionary Structured English Structure chart	Specification or analysis Design Coding and test Maintenance

Data structured systems development methodology (DSSD) [48]		
Data model Functions Results	Data structured diagram Warnier/Orr diagram Assembly-line diagram Entities diagram	Context definition Function definition Results definition

Jackson system development (JSD) [49]		
Initial model Functional model	Entity life history diagrams Structured English	Entity/action step Initial model step Interactive function step Information function step System timing step System implementation step

OMT methodology [12]		
Object model Dynamic model Functional model	Class and object diagram Modules diagram States diagram Process diagram Interaction diagram	Conceptualization Analysis Design Evolution

UML and RUP [19]		
Use case model Analysis model Design model Deployment model Implementation model Test model	Class diagram Use case diagram Interaction diagram State diagram Components diagram Activity diagram Components diagram Deployment diagram	Dynamic: inception, elaboration, construction, and transition Static: business modeling requirement, analysis and design, implementation, test, and deployment

Extreme programming [17]		
Software releases All SE techniques	Communication Feedback Simplicity Courage Respect	Coding Testing Listening Designing

Scrum [18]		
Software releases Meetings	Main roles: Scrum Master Product Owner Team	Sprint planning meeting Daily Scrum meeting Team work Sprint review meeting Sprint retrospective

**Table 3:** Knowledge engineering timeline.

Knowledge engineering			
Era	Periods	Description	Methodologies
Mastering intelligence (1956–1978)	General solvers		
	KBS	Knowledge	

Mastering process (1979–1989)	Process	Crisis. Knowledge engineering Shells	Buchanan KLIC IDEAL
	Specialization	Domain specific application	

Mastering complexity (1990–2000)	Second generation	KBS transfer Knowledge industry	MIKE Protégé CommonKADS
	Reusing	Tasks libraries Knowledge management	

Mastering communications (2001–2010)	Distributed	Ontologies Semantic Web	Mas-CK MASE W3C RDF METHONTOLOGY
	Data mining	Database availability Automatic learning	

Mastering productivity (2010–…)	Expanding Integration	Transfer to many domains Integrated approach	SKEngineering

**Table 4:** KBS development methodologies.

Artefact	Notation	Stages/workflows
Buchanan [26]		
Knowledge bases Inference methods	Rules Frames	Identification Conceptualization Formalization Implementation Validation

IDEAL [37]		
Plan Use case model Static conceptual model Process and control model Formal model Computational model	Rule languages LISP	Identification of the tasks Development of prototypes Execution of integrated system Perfective maintenance Technology transfer

CommonKADS [28]		
Organization model Task model Agent model Knowledge model Communication model Design model	Inference diagrams Task-methods diagrams Class diagrams State diagrams Use case diagrams Templates	Revision Risks study Monitoring

MIKE [29]		
Elicitation model KARL model Design model	KARL Design KARL	Acquisition Interpretation Formalization Design Implementation Evolution

Protégé [30]		
Knowledge model	Knowledge elicitation tool Knowledge base	—

METHONTOLOGY [31]		
Ontologies: concepts, relationships	Ontology languages: OIL DAML + OIL OWL	Predevelopment Development Postdevelopment Management and support

UPON [42]		
Domain and reference lexicon UML class diagrams UML activity diagrams Semantic network, ontology	OWL	Requirement Analysis Design Implementation
